# Diacylglycerol oil for the metabolic syndrome

**DOI:** 10.1186/1475-2891-6-43

**Published:** 2007-12-11

**Authors:** Hidekatsu Yanai, Yoshiharu Tomono, Kumie Ito, Nobuyuki Furutani, Hiroshi Yoshida, Norio Tada

**Affiliations:** 1 Department of Internal Medicine, The Jikei University School of Medicine, Chiba, Japan; 2 Department of Laboratory Medicine, The Jikei University School of Medicine, Chiba, Japan

## Abstract

Excess adiposity has been shown to play a crucial role in the development of the metabolic syndrome. The elevated fasting and postprandial triglyceride-rich lipoprotein levels is the central lipid abnormality observed in the metabolic syndrome. Recent studies have indicated that diacylglycerol (DAG) is effective for fasting and postprandial hyperlipidemia and preventing excess adiposity by increasing postprandial energy expenditure. We will here discuss the mechanisms of DAG-mediated improvements in hyperlipidemia and in postprandial energy expenditure, and effects of DAG oil on lipid/glucose metabolism and on body fat. Further, the therapeutic application of DAG for the metabolic syndrome will be considered.

## Introduction

Visceral fat accumulation has been shown to play a crucial role in the development of the metabolic syndrome, which is highly atherogenic. Dyslipidemia associated with the metabolic syndrome are elevated fasting and postprandial triglyceride (TG)-rich lipoproteins and decreased high-density lipoprotein (HDL) [1]. Insulin resistance resulting from obesity decreases lipoprotein lipase (LPL) activity, and then, a reduced LPL activity leads to the decreased clearance of fasting and postprandial TG-rich lipoproteins and to the decreased production of HDL [1]. The elevated level of fasting and postprandial TG-rich lipoproteins is the typical lipid abnormality observed in the metabolic syndrome [1].

Diacylglycerol (DAG) oil is present in edible vegetable oils. Recent studies have indicated that DAG is effective for fasting and postprandial hyperlipidemia and for preventing excess adiposity [2]. We will here discuss the therapeutic application of DAG for the metabolic syndrome.

## Biochemical properties of DAG

**Figure 1:**
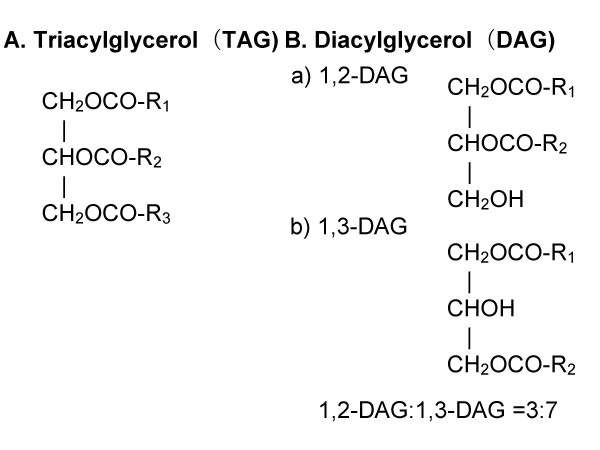
Structure of triacylglycerol and diacylglycerol. R1, R2, and R3 indicate fatty acids.

DAG is a natural component of various edible oils (Table 1) [3,4]. DAG can be synthesized enzymatically with the reverse reaction of 1,3-specific lipase, and consists mainly of the 1,3-species due to the migration of the acyl group in an equilibrium reaction. The ratio of the 1,3-DAG to 1,2-DAG in DAG oil is approximately 7:3 (Fig. 1) [2].

**Table 1:** Contents (weight %) of triacylglycerol and diacylglycerol in various edible oils [3,4]

	Triacylglycerol	Diacylglycerol
Soybean oil	97.9	1.0
Cottonseed oil	87.0	9.5
Palm oil	93.1	5.8
Corn oil	95.8	2.8
Safflower oil	96.0	2.1
Olive oil	93.3	5.5
Rapeseed oil	96.8	0.8
Lard	97.9	1.3

## The mechanism of DAG-mediated improvement in postprandial hyperlipidemia

**Figure 2:**
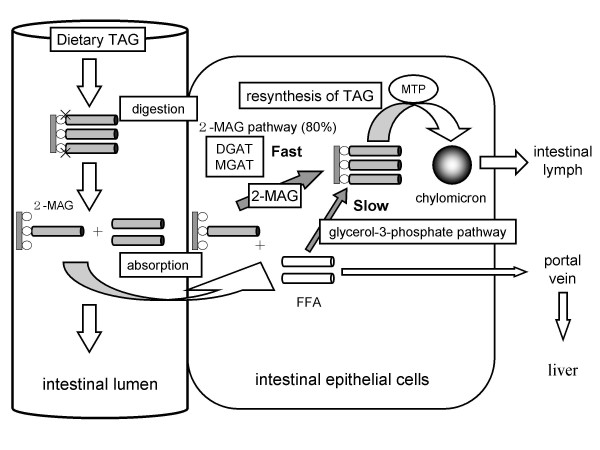
Digestion and absorption of triacylglycerol. DGAT, diacylglycerol acyltransferase; FFA, free fatty acids; 2-MAG, 2-monoacylglycerol; MGAT, monoacylglycerol acyltransferase; MTP, microsomal triglyceride transfer protein; TAG, triacylglycerol.

Dietary TAG oil is hydrolyzed by lipase to free fatty acids (FFA) and 2-monoacylglycerol in the small intestinal lumen, and these are absorbed by intestinal cells (Fig. 2). In intestinal cells, TG is re-synthesized from 2-monoacylglycerol and two FFA via the 2-monoacylglycerol pathway [5]. Monoacylglycerol acyltransferase (MGAT) and diacylglycerol acyltransferase (DGAT) work in the 2-monoacylglycerol pathway [6,7]. TG is incorporated into chylomicrons (CM) by microsomal triglyceride transfer protein (MTP), which are released into the intestinal lymph and poured into the bloodstream [8].

**Figure 3:**
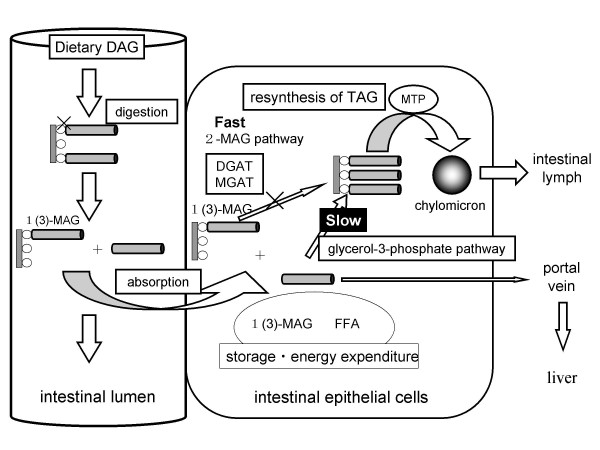
Digestion and absorption of diacylglycerol. DGAT, diacylglycerol acyltransferase; FFA, free fatty acids; 1(3)-MAG, 1-monoacylglycerol or 3-monoacylglycerol; MGAT, monoacylglycerol acyltransferase; MTP, microsomal triglyceride transfer protein; TAG, triacylglycerol.

In the case of DAG oil, the metabolic pathway in the intestinal cells is different from that of TAG oil (Fig. 3). Dietary DAG oil is mainly in the form of 1,3-DAG. 1,3-DAG would be hydrolyzed to initially to 1-monoacylglycerol and then, to glycerol and FFA, which are absorbed into the intestinal cells [9]. TG cannot be synthesized from 1-monoacylglycerol via the 2-monoacylglycerol pathway in the intestinal cells, because 1-monoacylglycerol cannot be the substrate for both DGAT and MGAT [6,7]. TG could be synthesized via the glycerol-3-phosphate pathway, which is less active than the 2-monoacylglycerol pathway [10]. 1,2-DAG would be hydrolyzed to 2-monoacylglycerol, and TG is synthesized via the 2-monoacylglycerol pathway [2].

Recently, Yasunaga K, et al found that DAG oil reduced plasma TG levels, resulting from more efficient clearance of DAG by both LPL-mediated lipolysis and apolipoprotein E-mediated hepatic endocytosis [11]. In also Sprague-Dawley rats, a lower plasma TG levels was accompanied by an increase in adipocyte LPL activity [12].

At present, slower re-acylation to TAG in small intestinal cells, an increase in LPL activity and apolipoprotein E-mediated hepatic endocytosis are supposed to be the underlying mechanisms improving the postprandial hyperlipidemia by substitution of DAG for TAG ingestion.

## Effects of DAG oil on lipid and glucose metabolism

An outline of reported effects of DAG on lipid/glucose metabolism is shown in Table 2. In animals, DAG ingestion was demonstrated to reduce plasma TG and FFA levels compared with TAG ingestion. Fujii A, et al found that DAG-rich oil reduced atherosclerosis in diabetic apoE-deficient mice, and ingestion of DAG-rich oil was associated with reduction in plasma cholesterol levels within larger TG-rich lipoproteins [13]. Further, DAG ingestion was reported to prevent the high-sucrose-diet-induced development of impaired glucose tolerance compared with TAG oil ingestion, in male Wistar rats [14].

**Table 2:** Effects of DAG on lipid and glucose metabolism

**fasting serum lipids [20,21,22]**
↓ triglyceride
↑ high-density lipoprotein cholesterol
↓ total cholesterol
↓ low-density lipoprotein cholesterol

**postprandial serum lipids [15,16,17]**

↓ triglyceride
↓ remnant-like lipoprotein particle triglyceride
↓ remnant-like lipoprotein particle cholesterol
↓ chylomicron triglyceride

**glucose metabolism [19]**

↓ hemoglobin A1c

In our previous studies with healthy volunteers, serum TG and remnant-like lipoprotein particles-cholesterol (RLP-C) concentrations after DAG ingestion were significantly lower than those after TAG ingestion [15]. Tomonobu K, et al. also reported that postprandial TG, RLP-C, and CM-TG concentrations were significantly lower after DAG ingestion than after TAG ingestion [16]. In our study with diabetic patients, DAG loading significantly suppressed increases in postprandial serum TG, RLP-C and RLP-TG levels as compared with TAG loading [17].

Recent study found that DAG reduced postprandial increase in TG, RLP-C, and RLP-TG, especially in subjects with insulin resistance [18]. In the subjects who consumed daily 10 g of DAG for 12 weeks, serum TG levels were decreased by 39.4%, and serum hemoglobin A1c levels were also decreased by 9.7%, compared with subjects who consumed TAG, suggesting that DAG ingestion also ameliorates glucose metabolism [19]. Further, a long-term DAG oil consumption has been reported to increase HDL-C, and decrease fasting TG, total cholesterol, LDL-C, compared with TAG consumption [20-22].

The apolipoprotein C-II is a cofactor of LPL, which hydrolyzes TG of CM and very-low-density lipoprotein (VLDL) [23]. We have a therapeutic experience with DAG oil to a patient with apolipoprotein C-II deficiency, a rare autosomal recessively-inherited disease [24]. In a patient with apolipoprotein C-II deficiency, DAG ingestion suppressed increase in serum TG, VLDL-C, and RLP-C levels compared with TAG ingestion, suggesting that DAG can decrease TG-rich lipoprotein, also independent of LPL.

In summary, DAG ameliorates fasting and postprandial TG-rich lipoproteins, and glucose metabolism, which may be favorable for metabolic disorders observed in the metabolic syndrome.

## The mechanism to promote negative caloric balance by DAG ingestion

Compared with the TAG-containing meal, the DAG-containing meal tended to induce higher postprandial energy expenditure and significantly lower postprandial respiratory quotient, suggesting that the DAG-containing meal has high postprandial lipid oxidation activity and a potential effect on high diet-induced thermogenesis [25,26]. Upregulated mRNA expressions associated with FA transport (FA translocase and FA binding protein), β-oxidation (acyl-CoA oxidase and medium-chain acyl-CoA dehydrogenase), and thermogenesis (uncoupling protein-2) in the small intestine by DAG may explain in part mechanisms for increased postprandial energy expenditure [27,28].

## Effect of a long-term consumption of dietary DAG for adiposity

An outline of a long-term effect of DAG ingestion on body composition is shown in Table 3. In rats, DAG-rich oil ingestion was effective in suppressing FA synthase activity and enhancing β-oxidation activity, reducing the abdominal fat [29]. In brown adipose tissue-deficient mice, a model of high-fat diet-induced insulin resistance and obesity, a long term substitution of DAG for TAG reduced Western-type diets induced insulin resistance and body fat accumulation by suppressing hepatic gluconeogenesis and stimulating fat oxidation in skeletal muscle [30].

**Table 3:** A long-term effect of DAG ingestion on body composition [20,21,22,31,32,33]

↓ body weight
↓ body fat
↓ Visceral fat
↓ Subcutaneous fat
↓ hepatic fat
↓ Waist circumferences
↓ Skin fold thickness

A long-term consumption of DAG decreased body fat, especially visceral fat, and decreased body weight in both overweight and normal Japanese people, and obese subjects in the United States, compared with TAG consumption [31-33]. Open-labeled long-term consumption study indicated that DAG decreased body weight compared with TAG consumption [20]. In several long-term studies, decrease in waist circumferences and skin fold thickness by DAG consumption were observed [21,22,33].

Thus, several long-term clinical trials have indicated that DAG consumption results in losses of body weight and body fat, in healthy non-obese and obese men and women. Further, DAG oil ingestion decreased both the abdominal fat area and leptin in obese children, suggesting that DAG oil prevents excess adiposity in children as well as in adults [34]. Dairy ingestion of 8–20 g DAG has been used in human studies on the effect of DAG on body composition [35]. However, it remains unknown how dose DAG is high effective for preventing excess adiposity, which should be investigated in the future.

In summary, DAG may be beneficial in preventing excess adiposity, which may be favorable for the metabolic syndrome.

## A safety of a chronic consumption of DAG

In c-Ha-ras proto-oncogene transgenic rats, DAG oil administration was associated with a significant increase in the incidence of squamous cell carcinomas of the tongue with the Cochran-Armitage trend test and also number of tumors in coefficients for linear contrast trend tests [36]. However, 24 months-DAG-treated rats had no higher risk of carcinogenic effects than rats fed on similar feeding regimens with TAG [37]. The potential chronic toxic effects of DAG when administered orally for 12 months were evaluated using Beagle dogs [38]. DAG at dietary concentrations up to 9.5% for one year had no effect on normal dog growth and development, in comparison to TAG [38]. In mice, DAG at dietary concentrations up to 6.0% for 24 months produced no signs of systemic toxicity and had no effect on the incidence of neoplastic findings [39]. Most of studies investigating a chronic dietary toxicity of DAG reported that DAG did not produce systemic toxicity and had no effect on the incidence of neoplastic findings. However, we should observe the safety of a chronic consumption of DAG carefully.

## Conclusion

DAG oil consumption has been reported to ameliorate the constituents of the metabolic syndrome such as excess adiposity, impaired glucose metabolism, and dyslipidemia, suggesting the usefulness of DAG oil for the management and prevention of the metabolic syndrome.
